# Fish consumption and the risk of colorectal cancer: the Ohsaki Cohort Study

**DOI:** 10.1038/sj.bjc.6605217

**Published:** 2009-07-28

**Authors:** Y Sugawara, S Kuriyama, M Kakizaki, M Nagai, K Ohmori-Matsuda, T Sone, A Hozawa, Y Nishino, I Tsuji

**Affiliations:** 1 Division of Epidemiology, Department of Public Health and Forensic Medicine, Tohoku University Graduate School of Medicine, Sendai, Japan; 2 Division of Epidemiology, Miyagi Prefectural Cancer Research Center, Natori, Japan

**Keywords:** fish consumption, colorectal cancer, incidence, prospective cohort study, Japan

## Abstract

**Background:**

Evidence from laboratory and animal studies suggests that high fish consumption may reduce the risk of colorectal cancer, but the results of studies in humans have been inconsistent. The objective of this study was to prospectively examine the association between fish consumption and the risk of colorectal cancer incidence in Japan, where fish is widely consumed.

**Methods:**

We analysed data from 39 498 men and women registered in the Ohsaki National Health Insurance Cohort Study who were 40–79 years old and free of cancer at the baseline. Fish consumption was assessed at the baseline using a self-administered food frequency questionnaire.

**Results:**

During 9 years of follow-up, we identified 566 incident cases of colorectal cancer (379 men and 187 women). The hazard ratios and 95% confidence intervals (CIs) for colorectal cancer incidence in the highest quartile of fish consumption compared with the lowest quartile were 1.07 (95% CIs; 0.78–1.46, *P*-trend=0.43) for men, and 0.96 (95% CIs; 0.61–1.53, *P*-trend=0.69) for women.

**Conclusion:**

The results of this prospective cohort study revealed no association between fish consumption and the risk of colorectal cancer.

In association with marked changes in dietary habits over the last few decades, the incidence of colorectal cancer has been increasing in Japan. In 2002, the world age-standardised incidence rate of colorectal cancer was the second commonest cancer in men (70.7 per 100 000) and the commonest cancer in women (38.6 per 100 000) (Matsuda *et al*, 2008).

Evidence has suggested associations between consumption of red meat, vegetables, and fruit, and colorectal cancer incidence, but any association with fish consumption is still unclear (World Cancer Research Fund, 2007).

Although laboratory and animal studies suggest that consumption of fish, rich in n-3 fatty acids, inhibits carcinogenesis (Takahashi *et al*, 1997; Rao *et al*, 2001), the results of studies in humans have been inconsistent. Most of the previous studies were conducted in countries where fish consumption is relatively low. Japan has the highest fish consumption among the developed countries (Food Balance Sheet, 2003), but only one prospective study has examined the association with colorectal cancer risk in Japan (Kobayashi *et al*, 2004). Therefore, we analysed data from a large population-based prospective cohort study in Japan.

## Materials and methods

We used data from the Ohsaki National Health Insurance (NHI) Cohort Study, the design of which has been described in detail elsewhere (Tsuji *et al*, 1998; Kuriyama *et al*, 2006). Briefly, between October and December 1994, we delivered a self-administered 40-item food frequency questionnaire (FFQ) to men and women aged 40–79 years old living in the catchment area of Ohsaki Public Health Center, a local government agency providing preventive health services in 14 municipalities in Miyagi Prefecture, northeastern Japan. Among 54 996 eligible individuals, 52 029 responded to the questionnaire (94.6%), but 776 who had withdrawn from the NHI before the baseline questionnaire survey were excluded, leaving 51 253 (24 573 men and 26 680 women) in the study cohort. The study protocol was approved by the institutional review board of the Tohoku University School of Medicine. We considered the return of the questionnaire to imply the participant’s consent to participate in the study.

The self-administrated questionnaire also covered the personal and family history of diseases, and the lifestyle factors such as smoking status, alcohol consumption, time spent walking per day, job status, marital status, and education. In the FFQ, there were three items relating to fish (i.e., fresh fish, fish products, and dried or salted fish). Fish products were mostly fish paste made from boiled white fish meat (known as ‘kamaboko’ and ‘chikuwa’ in Japan). Frequency was in five categories: almost never, 1–2 days per month, 1–2 days per week, 3–4 days per week, or almost every day. For seasonal foods, participants were asked to report their intake in the high season. We also collected 12-day dietary records from a subsample of the participants, and determined the portion size for each food item based on the median values in the records. The validity of fresh fish and fish product consumption assessed by the FFQ has been reported earlier (Ogawa *et al*, 2003). Spearman's correlation coefficients for fresh fish consumption were 0.39 for men and 0.60 for women, and those for fish product consumption were 0.49 for men and 0.41 for women. We did not assess dried or salted fish consumption because the Spearman correlation coefficients indicated a negative correlation. We calculated the weight of total fish consumed each day on the basis of the two items: fresh fish and fish products by multiplying the frequency by the portion size for each fish item; for fresh fish, 95.0 g day^−1^ in men and 80.0 g day^−1^ in women, and for fish products, 27.5 g day^−1^ in men and 26.7 g day^−1^ in women, and summing the two items.

We followed up the participants from 1 June 1995 to 31 December 2003. The end point was diagnosis of colorectal cancer, and the end of follow-up death, emigration, or end of the follow-up period, whichever occurred first. We collected withdrawals from the NHI because of death, emigration, and loss of NHI qualification from the NHI withdrawal history files. We also ascertained incident cases of cancer by computer link with the Miyagi Prefectural Cancer Registry, which covers the study area. In this registry, the percentages registered by death certificates only for colorectal cancer were 7.4% for men and 11.5% for women during 1998–2002 (Curado *et al*, 2007). Cancers were coded according to the International Classification of Diseases for Oncology, 2nd version (ICD-O-2) as colon cancer (C18.2-C18.9) and rectal cancer for the recto–sigmoid junction or rectum combined (C19.0-C20.9), so colorectal cancer was C18.2-C20.9. Furthermore, using hospital records, we classified cancers as localised (within the colon and rectum) or advanced (spread beyond or metastatic involving lymph nodes or other organs).

### Statistical analysis

We excluded participants who had a history of cancer (*N*=3155), who did not report on fish consumption (*N*=8202), and who reported extreme values of total daily caloric intake (sex-specific cutoffs for highest 0.5% and lowest 0.5%; *N*=398); this left 39 498 participants (18 858 men and 20 640 women) for this study.

We divided the participants into quartiles on the basis of the weight of total fish consumed (in grams) per day and examined its association with colorectal cancer risk. Person-years of follow-up were estimated for each participant from 1 June 1995 to the end of follow-up. Cox proportional hazards regression analysis was used to estimate the hazard ratios (HRs) and 95% confidence intervals (CIs) for colorectal cancer incidence according to the lowest quartile of total fish consumption as the reference group, after adjustment for potential confounders. We then divided the participants into tertiles on the basis of the total weight of fish consumed per day and examined the association with total fish consumption.

We also calculated the association between consumption of fresh fish and colorectal cancer incidence. Moreover, we examined the incidence of colorectal cancer classified according to clinical stage (localised or advanced). Colon cancer was also further classified by subsite: ascending (C18.2), transverse (C18.4), descending (C18.6), and sigmoid (C18.7), and the incidence calculated for each site.

We considered the following variables as potential confounders: age (continuous variable), body mass index in kg m^−2^ (<18.5, 18.5–24.9, or ⩾25.0), family history of cancer (yes or no), history of stroke (yes or no), history of hypertension (yes or no), history of myocardial infarction (yes or no), history of diabetes mellitus (yes or no), education (junior high school or less, high school, or college/university or higher), marital status (married or unmarried), job status (employed or unemployed), smoking status (never smoked, smoked in the past, currently smoking <20 cigarettes day^−1^, or ⩾20 cigarettes day^−1^), alcohol consumption (never drank alcohol, drank in the past, currently drinking), time spent walking (⩽0.5 h day^−1^, 0.5–1.0 h day^−1^, or ⩾1 h day^−1^), total caloric intake (continuous variable, kcal day^−1^), meat consumption (continuous variable, g day^−1^), vegetable consumption (continuous variable, g day^−1^), and fruit consumption (continuous variable, g day^−1^). The *P*-values for the test of linear trend (*P*-trend) were estimated using the weight of fish (in grams) consumed per day as a continuous variable. The effect modification between total fish consumption and all confounders was tested through the addition of cross-product terms to the multivariate model. All *P*-values were two-sided, and differences at *P*<0.05 were considered statistically significant. All statistical analyses were performed using the SAS statistical software package, version 9.1 (SAS Institute Inc., Cary, NC, USA).

## Results

Table 1 shows the baseline characteristics of the participants according to the quartiles of total fish consumption. Participants with higher fish consumption tended to be older, more likely to walk for more than 1 h, and to have a higher total caloric intake, including meat, vegetables, and fruit. In addition, more than 70% of men drank alcohol, and over 50% of men were current smokers. In contrast, 70% of women had never drunk alcohol or smoked. More men than women had a history of stroke, myocardial infarction, or diabetes mellitus, whereas more women than men had a history of hypertension.

During 305 894 person-years of follow-up, 566 incident cases of colorectal cancer (379 men and 187 women) were observed. Table 2 shows the HRs and 95% CIs of colorectal cancer according to the quartiles of total fish consumption in both men and women. Multivariate analysis showed that total fish consumption was not associated with colorectal cancer incidence in either men or women. The multivariate HRs (95% CIs) for men in the other groups *vs* the lowest quartile group were 1.04 (0.79–1.39), 1.11 (0.81–1.53), and 1.07 (0.78–1.46), respectively (*P*-trend=0.43), whereas those for women were 1.19 (0.79–1.81), 1.17 (0.73–1.88), and 0.96 (0.61–1.53), respectively (*P*-trend=0.69). Furthermore, separate analyses for colon and rectal cancer, as well as for colorectal cancer overall, showed no association with total fish consumption. For colon cancer, the HRs (95% CIs) for men and women in the highest fish consumption group were 1.11 (0.75–1.64; *P*-trend=0.27) and 0.95 (0.53–1.71; *P*-trend=0.75), respectively. For rectal cancer, the corresponding values were 0.99 (0.61–1.61; *P*-trend=0.95) and 0.96 (0.47–1.96; *P*-trend=0.80), respectively. When total fish consumption was divided into tertiles, no association was observed between total fish consumption and colorectal cancer. The multivariate HRs (95% CIs) for men in the highest *vs* the other tertiles were 1.10 (0.86–1.41) and 1.08 (0.80–1.46) (*P*-trend=0.43), and the corresponding values for women 1.06 (0.73–1.55) and 0.94 (0.63–1.42) (*P*-trend=0.69).

Similarly, no association with fresh fish consumption was observed. The multivariate HRs (95% CIs) for men in the other quartiles *vs* the lowest quartile were 0.88 (0.53–1.46), 0.93 (0.57–1.53), and 1.03 (0.62–1.69), respectively (*P*-trend=0.33), whereas the corresponding values for women were 1.58 (0.67–3.76), 1.54 (0.66–3.60), and 1.32 (0.56–3.15), respectively (*P*-trend=0.54). The result was the same when we conducted separate analyses for colon and rectal cancers (data not shown). No significant associations were observed when colorectal cancer was classified by clinical stage. For localised disease, the HR (95% CIs) for men in the highest group was 1.12 (0.72–1.75; *P*-trend=0.92) and that for women was 0.99 (0.50–1.98; *P*-trend=0.61). For advanced colorectal cancer, the HR (95% CIs) for men in the highest group was 0.91 (0.58–1.44; *P*-trend=0.90) and that for women was 0.73 (0.38–1.39; *P*-trend=0.35). When examined by colon cancer subsite, no association with total fish consumption was observed for any subsite (data not shown).

All trends remained similar after exclusion of colorectal cancer cases that had been diagnosed during the first 3 years of follow-up. We also conducted stratified analyses according to age and other potential confounders but found no associations with total fish consumption (data not shown). Furthermore, effect modification was tested with all confounders, but significant effect modifications were observed only with total fish consumption.

## Discussion

In this population-based cohort study in Japan, we found no significant associations between fish consumption and the incidence of colorectal cancer. In the 20 prospective studies that have examined this question, eight reported an inverse (Willett *et al*, 1990; Bostick *et al*, 1994; Goldbohm *et al*, 1994; Gaard *et al*, 1996; Kato *et al*, 1997; Tiemersma *et al*, 2002; Norat *et al*, 2005; Hall *et al*, 2008), 1 reported a positive (Knekt *et al*, 1999), and the remaining 11 reported no association (Giovannucci *et al*, 1994; Pietinen *et al*, 1999; Ma *et al*, 2001; English *et al*, 2004; Kobayashi *et al*, 2004; Lin *et al*, 2004; Sanjoaquin *et al*, 2004; Larsson *et al*, 2005; Lüchtenborg *et al*, 2005; Engeset *et al*, 2007; Lee *et al*, 2009). Our result is consistent with the latter 11 studies.

In only three of the eight studies reporting an inverse association was this statistically significant (Kato *et al*, 1997; Norat *et al*, 2005; Hall *et al*, 2008). There are several possible reasons for the discrepancy between our finding and the latter three studies. First, the weight of fish consumed daily differed among the study populations. In fact, this was only about one-third of that in our study (Food Balance Sheet, 2003). Second, the fish species consumed would have differed among the study populations. In our study there was a high intake of tuna, mackerel, and sardine, which contain high levels of n-3 fatty acids, whereas the other studies had higher intakes of salmon, cod, and herring (Food Balance Sheet, 2003). If the results were influenced by fish species, the association between fish consumption and the incidence of colorectal cancer might appear more strongly in our study population because our study population had a high intake of fish which contain high levels of n-3 fatty acids. However, we observed no association with total fish consumption, suggesting that the difference in fish species did not explain the discrepancy with other studies. Third, high fish consumers in Western countries may be more health-conscious individuals, and as there was no adjustment for potential confounding by vegetable or fruit consumption, this remains a possibility.

One study from Finland reported an increased incidence of colon cancer that was attributed to smoked and salted fish (Knekt *et al*, 1999), but the cooking methods may have influenced the results.

Our study had several strengths including recruitment of individuals from the general population and the occurrence of a larger number of colorectal cancer cases (566) than recent prospective studies. Second, the response rate was high (94.6%) and the selection bias was small. Third, our study population was in Japan, which has the highest fish consumption in the world (Food Balance Sheet, 2003).

The first of the limitations is that we collected information on fish consumption frequency only once and by a self-reported questionnaire. Therefore, some misclassification of exposure would have been inevitable, and the reported HR was likely to have underestimated the true relationship, although the questionnaire was validated (Ogawa *et al*, 2003). Second, dried or salted fish consumption was not included in our estimate of total fish consumption, so actual total fish consumption may have been underestimated. However, the portion sizes of dried or salted fish calculated by a validated study were small (10 g day^−1^, among both men and women). Third, we were unable to check whether the use of aspirin or anti-inflammatory drugs influenced the association with fish consumption (Chan *et al*, 2005). However, as the Japanese hardly ever use aspirin or anti-inflammatory drugs on a prophylactic basis, the probability of such confounding was probably low.

In conclusion, this prospective study has found that total fish consumption is not associated with the incidence of colorectal cancer in Japan.

## Figures and Tables

**Table 1:** Characteristics of participants according to fish consumption

	**Quartile of total fish consumption**			
	**1 (low)**	**2**	**3**	**4 (high)**
*Men, g day* ^−1^	0–26.2	26.3–53.3	53.4–96.3	⩾96.4
Number of participants	5081	5611	3336	4830
Age, years (s.d.)	56.6 (11.3)	58.0 (10.7)	59.3 (10.1)	59.6 (9.7)
Body mass index, kg m^−2^ (s.d.)	23.3 (3.2)	23.5 (2.9)	23.4 (3.0)	23.5 (2.9)
Family history of cancer (%)	28.6	31.3	31.5	32.1
History of stroke (%)	2.5	2.1	2.7	2.3
History of hypertension (%)	21.1	23.8	23.2	26.4
History of myocardial infaction (%)	3.0	2.7	3.4	3.0
History of diabetes mellitus (%)	6.8	7.2	7.7	8.0
Education (%)				
Junior high school or less	49.0	50.2	56.2	58.5
High school	39.0	39.6	34.8	34.4
College/university or higher	12.0	10.2	9.0	7.1
Marital status (%)				
Married	86.5	89.8	91.2	92.2
Unmarried	13.5	10.2	8.8	7.8
Job status (%)				
Employed	80.0	79.6	78.9	81.8
Unemployed	20.0	20.4	21.1	18.2
Smoking status (%)				
Never smoked	17.4	20.3	19.4	22.1
Smoked in the past	23.8	27.5	26.6	28.5
Currently smoking, <20 cigarettes day^−1^	17.4	15.5	17.2	17.2
Currently smoking, ⩾20 cigarettes day^−1^	41.4	36.7	36.8	32.2
Alcohol consumption (%)				
Never drank alcohol	18.8	15.5	14.9	13.4
Drank in the past	9.8	9.7	9.9	8.8
Currently drinking	71.4	74.8	75.2	77.8
Time spent walking (%)				
⩽0.5 h day^−1^	33.6	28.4	26.9	25.0
0.5–1.0 h day^−1^	25.0	25.7	25.1	23.4
⩾1 h day^−1^	41.4	45.9	48.0	51.6
Total caloric intake, kcal (s.d.)	1645.8 (562.4)	1832.6 (557.2)	1931.7 (577.9)	2083.7 (579.7)
Meat consumption, g (s.d.)	18.2 (13.9)	21.6 (14.6)	22.0 (18.1)	27.3 (20.7)
Vegetable consumption, g (s.d.)	36.1 (28.2)	48.0 (29.8)	55.0 (34.0)	65.2 (35.2)
Fruit consumption, g (s.d.)	57.7 (50.3)	76.0 (52.8)	83.1 (56.6)	96.8 (57.2)
				
*Women, g day* ^−*1*^	0–26.6	26.7–45.7	45.8–81.3	⩾81.4
Number of participants	4752	6259	3611	6018
Age, years (s.d.)	59.6 (11.0)	59.3 (10.4)	60.7 (9.9)	60.8 (9.2)
Body mass index, kg m^−2^ (s.d.)	23.7 (3.6)	23.7 (3.3)	23.7 (3.2)	23.9 (3.3)
Family history of cancer (%)	32.1	34.4	35.2	34.3
History of stroke (%)	2.0	1.4	0.7	1.0
History of hypertension (%)	25.2	26.8	26.6	28.5
History of myocardial infaction (%)	1.7	1.6	1.7	2.3
History of diabetes mellitus (%)	5.4	5.0	5.5	5.1
Education (%)				
Junior high school or less	52.7	45.8	49.6	49.3
High school	36.4	43.2	39.4	41.3
College/university or higher	10.9	11.0	11.0	9.4
Marital status (%)				
Married	71.1	76.5	78.2	79.3
Unmarried	28.9	23.5	21.8	20.7
Job status (%)				
Employed	44.9	47.0	44.4	47.9
Unemployed	55.1	53.0	55.6	52.1
Smoking status (%)				
Never smoked	85.7	90.2	91.3	94.0
Smoked in the past	2.8	2.7	1.9	2.0
Currently smoking, <20 cigarettes day^−1^	7.4	4.8	4.7	2.7
Currently smoking, ⩾20 cigarettes day^−1^	4.1	2.3	2.1	1.3
Alcohol consumption (%)				
Never drank alcohol	70.1	71.7	73.0	76.0
Drank in the past	5.7	4.5	3.6	3.1
Currently drinking	24.2	23.8	23.4	20.9
Time spent walking (%)				
⩽0.5 h day^−1^	34.5	30.0	29.8	25.8
0.5–1.0 h day^−1^	28.2	29.3	28.2	28.8
⩾1 h day^−1^	37.3	40.7	42.0	45.4
Total caloric intake, kcal (s.d.)	1100.3 (315.1)	1253.8 (299.2)	1326.6 (298.9)	1437.2 (306.7)
Meat consumption, g (s.d.)	12.9 (10.6)	16.3 (11.1)	15.5 (12.8)	18.2 (13.7)
Vegetable consumption, g (s.d.)	46.6 (33.8)	61.6 (34.3)	67.7 (37.2)	76.9 (38.4)
Fruit consumption, g (s.d.)	92.9 (63.8)	116.6 (59.7)	125.3 (61.4)	138.6 (58.2)

s.d.=standard deviation.

**Table 2:** Hazard ratios (HRs) and 95% confidence intervals (CIs) for colorectal cancer incidence according to total fish consumption: the Ohsaki Cohort Study

	**Quartile of total fish consumption**				
	**1 (low)**	**2**	**3**	**4 (high)**	***P*-trend^a^**
*Men, g day* ^−*1*^	0–26.2	26.3–53.3	53.4–96.3	⩾96.4	
Colorectal Cancer					
Person-years of follow-up	39 264	43 517	25 922	37 065	
Number of cases	90	112	74	103	
Age-adjusted HR (95% CI)	1.00 (reference)	1.06 (0.81–1.40)	1.12 (0.83–1.53)	1.09 (0.82–1.44)	0.36
Multivariable HR^b^ (95% CI)	1.00 (reference)	1.04 (0.79–1.39)	1.11 (0.81–1.53)	1.07 (0.78–1.46)	0.43
					
Colon cancer					
Person-years of follow-up	39 362	43 662	26 024	37 180	
Number of cases	55	64	42	68	
Age-adjusted HR (95% CI)	1.00 (reference)	0.98 (0.69–1.41)	1.03 (0.69–1.54)	1.16 (0.82–1.66)	0.17
Multivariable HR^b^ (95% CI)	1.00 (reference)	0.95 (0.66–1.38)	0.99 (0.65–1.50)	1.11 (0.75–1.64)	0.27
					
Rectal cancer					
Person-years of follow-up	39 427	43 703	26 033	37 267	
Number of cases	38	50	35	40	
Age-adjusted HR (95% CI)	1.00 (reference)	1.14 (0.74–1.73)	1.29 (0.81–2.04)	1.02 (0.66–1.60)	0.94
Multivariable HR^b^ (95% CI)	1.00 (reference)	1.12 (0.73–1.73)	1.28 (0.80–2.06)	0.99 (0.61–1.61)	0.95
					
*Women, g day* ^−*1*^	0–26.6	26.7–45.7	45.8–81.3	⩾81.4	
Colorectal Cancer					
Person-years of follow-up	36 160	48 673	28 195	47 098	
Number of cases	38	61	37	51	
Age-adjusted HR (95% CI)	1.00 (reference)	1.21 (0.81–1.82)	1.22 (0.77–1.91)	1.00 (0.66–1.53)	0.87
Multivariable HR^b^ (95% CI)	1.00 (reference)	1.19 (0.79–1.81)	1.17 (0.73–1.88)	0.96 (0.61–1.53)	0.69
					
Colon cancer					
Person-years of follow-up	36 185	48 766	28 219	47 166	
Number of cases	24	38	25	31	
Age-adjusted HR (95% CI)	1.00 (reference)	1.20 (0.72–2.00)	1.30 (0.74–2.27)	0.96 (0.57–1.64)	0.83
Multivariable HR^b^ (95% CI)	1.00 (reference)	1.19 (0.71–2.00)	1.25 (0.70–2.24)	0.95 (0.53–1.71)	0.75
					
Rectal cancer					
Person-years of follow-up	36 217	48 788	28 282	47 178	
Number of cases	16	23	12	22	
Age-adjusted HR (95% CI)	1.00 (reference)	1.08 (0.57–2.03)	0.95 (0.45–2.00)	1.04 (0.56–1.98)	1.00
Multivariable HR^b^ (95% CI)	1.00 (reference)	1.04 (0.54–2.00)	0.91 (0.42–1.98)	0.96 (0.47–1.96)	0.80

a *P*-trend values were calculated using fish consumption per day as continuous variable.

b Multivariate HR was adjusted for age (continuous variable), body mass index in kg m^−2^ (<18.5, 18.5–24.9, or ⩾25.0), family history of cancer (yes or no), history of stroke (yes or no), history of hypertension (yes or no), history of myocardial infarction (yes or no), and history of diabetes mellitus (yes or no), education (junior high school or less, high school, or college/university or higher), marital status (married, or unmarried), job status (employed, or unemployed), smoking status (never smoked, smoked in the past, currently smoking <20 cigarettes day^−1^, or currently smoking ⩾20 cigarettes day^−1^), alcohol consumption (never drank alcohol, drank in the past, or currently drinking), time spent walking (⩽0.5 h day^−1^, 0.5–1.0 h day^−1^, or ⩾1 h day^−1^), total calorie intake (continuous variable, kcal day^−1^), meat consumption (continuous variable, g day^−1^), vegetable consumption (continuous variable, g day^−1^), fruit consumption (continuous variable, g day^−1^).
